# Foveated near-eye display using computational holography

**DOI:** 10.1038/s41598-020-71986-9

**Published:** 2020-09-10

**Authors:** Ali Cem, M. Kivanc Hedili, Erdem Ulusoy, Hakan Urey

**Affiliations:** Optical Microsystems Laboratory, Electrical and Electronics Engineering Department, Koç University, 34450 Istanbul, Turkey

**Keywords:** Displays, Imaging and sensing

## Abstract

Holographic display is the only technology that can offer true 3D with all the required depth cues. Holographic head-worn displays (HWD) can provide continuous depth planes with the correct stereoscopic disparity for a comfortable 3D experience. Existing HWD approaches have small field-of-view (FOV) and small exit pupil size, which are limited by the spatial light modulator (SLM). Conventional holographic HWDs are limited to about 20° × 11° FOV using a 4 K SLM panel and have fixed FOV. We present a new optical architecture that can overcome those limitations and substantially extend the FOV supported by the SLM. Our architecture, which does not contain any moving parts, automatically follows the gaze of the viewer’s pupil. Moreover, it mimics human vision by providing varying resolution across the FOV resulting in better utilization of the available space-bandwidth product of the SLM. We propose a system that can provide 28° × 28° instantaneous FOV within an extended FOV (the field of view that is covered by steering the instantaneous FOV in space) of 60° × 40° using a 4 K SLM, effectively providing a total enhancement of > 3 × in instantaneous FOV area, > 10 × in extended FOV area and the space-bandwidth product. We demonstrated 20° × 20° instantaneous FOV and 40° × 20° extended FOV in the experiments.

## Introduction

Head-worn displays (HWDs) have been gathering more and more attention over the last decade, especially due to the increasing popularity of augmented reality and virtual reality applications^1^. The majority of HWDs are fixed focus stereoscopic systems^2,3^, which results in a mismatch between the accommodation (focus) and vergence (rotation of the eyes to maintain single binocular vision) responses and inevitably leads to viewing discomfort^4,5^.

Holographic HWDs, on the other hand, show computer generated holograms (CGHs) with the correct perspective and all the natural depth cues to each eye by emulating the wavefront pattern that would be emitted from the displayed 3D objects^6,7^. As a result, the user can focus directly on the gazed 3D object, eliminating the discomfort caused by the vergence-accommodation conflict. In holographic HWDs, CGHs are typically displayed on phase-only spatial light modulators (SLMs)^8^. The need for relay optics is not present for such displays, since the SLM can be imaged at an arbitrary distance away from the eye^9^.

Despite their great potential, current holographic HWDs suffer from field-of-view (FOV) and eyebox (exit pupil) limitations, due to the limited pixel counts of currently available SLMs. Total pixel count of the SLM puts an upper limit on the space-bandwidth product (SBP) of the system^10–13^. To alleviate the SBP limitations, holographic displays can utilize pupil tracking and deliver the object waves through two small eyeboxes around the eye pupil, which maximizes the FOV attainable from the system^14^. State-of-the-art SLMs can have resolutions of up to 4 K (3,840 horizontal and 2,160 vertical pixels), a display designed with such an SLM would have a limited SBP (= product of FOV and eyebox size) of 100 deg × mm in the horizontal axis. As an example, one can obtain a horizontal FOV of 20 degrees and an eyebox size of 5 mm. A FOV of 20 degrees is small for a true AR experience and a 5 mm static (non-moving) eyebox is also small for a comfortable viewing experience using a binocular HWD^15^. In order to match human vision capabilities, one might desire a FOV of 80 degrees and an eyebox size of 15 mm, which will require an SLM with pixel count that is in the order of 50,000 pixels in the horizontal axis (more than gigapixels in total). Such an SLM is beyond the capabilities of current technology and would be overkill from the system design perspective. Various methods have been attempted to increase the eyebox size and the FOV simultaneously with state-of-the-art SLMs, including time multiplexing^16,17^ and using multiple SLMs for spatial multiplexing^18–20^. Despite their advantages over using a single SLM, these approaches result in complex and high-cost systems.

Since it is not currently possible to rival the FOV of human vision using holographic displays, one solution is to implement a foveated display where only the CGH for the gazed object is being generated and the peripheral image is displayed with a low-resolution microdisplay^14^. Using the properties of the human visual system is common practice in other display technologies, such as light field displays^21,22^. It is possible to combine a travelling microdisplay with a wide field-of-view peripheral display and enable natural focus depth using either a light field or a multifocal/varifocal approach^23^. Various methods have been attempted to steer the FOV from one region to another, which generally involve utilizing motorized optical components. Such solutions suffer from actuator size and synchronization issues between multiple dynamic elements, as it is not possible to steer the FOV with a single dynamic component.

**Figure 1:**
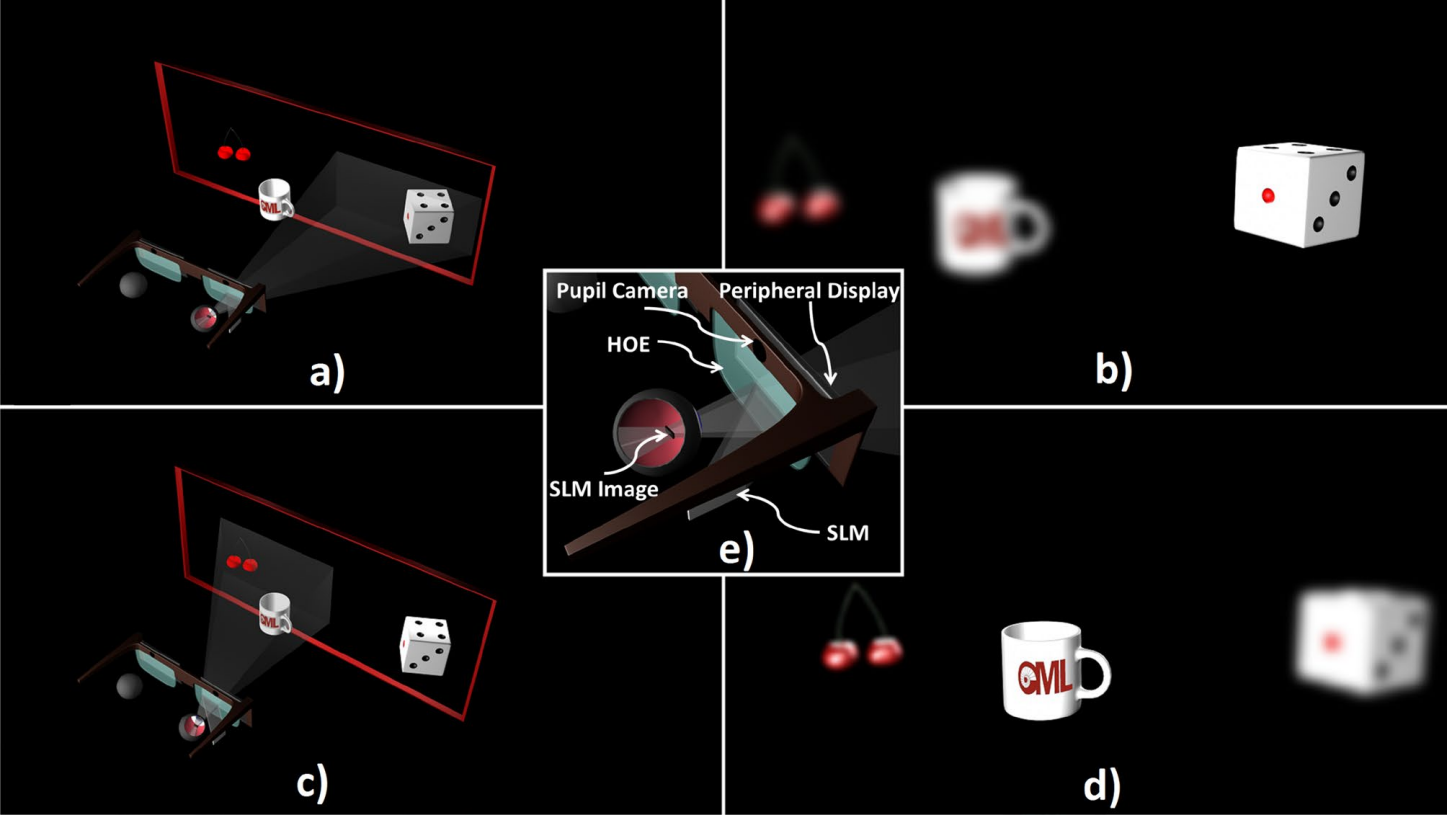
Foveated holographic display with the eye-centric design concept in eyeglass format. (**a**) Red box shows the total FOV that can be delivered by the AR display. Within the FOV, an SLM is used to deliver high-resolution holographic images to the fovea. The peripheral content is delivered by a low-resolution microdisplay (e.g., micro-LED or OLED on transparent substrate) that can be placed on the eyeglass frame. (**b**) The high-resolution holographic image that is projected to the fovea appears sharp and the peripheral image appears blurred. (**c**) The eye-centric design ensures that the SLM image follows the fovea for all gaze directions by imaging the SLM to the rotation center of the eye. (**d**) Eye rotations are measured in real-time with a pupil tracker to update the hologram on the SLM and content on the peripheral display accordingly. (**e**) A close-up illustration of the eye-centric design.

We propose a novel optical architecture for holographic HWD where the SLM is imaged at the rotation center of the eye. In this architecture, instantaneous FOV can be steered without using any dynamic optical components. A particular implementation of such a system in eyeglasses form factor is illustrated in Fig. 1.

## Eye-centric design concept

**Figure 2:**
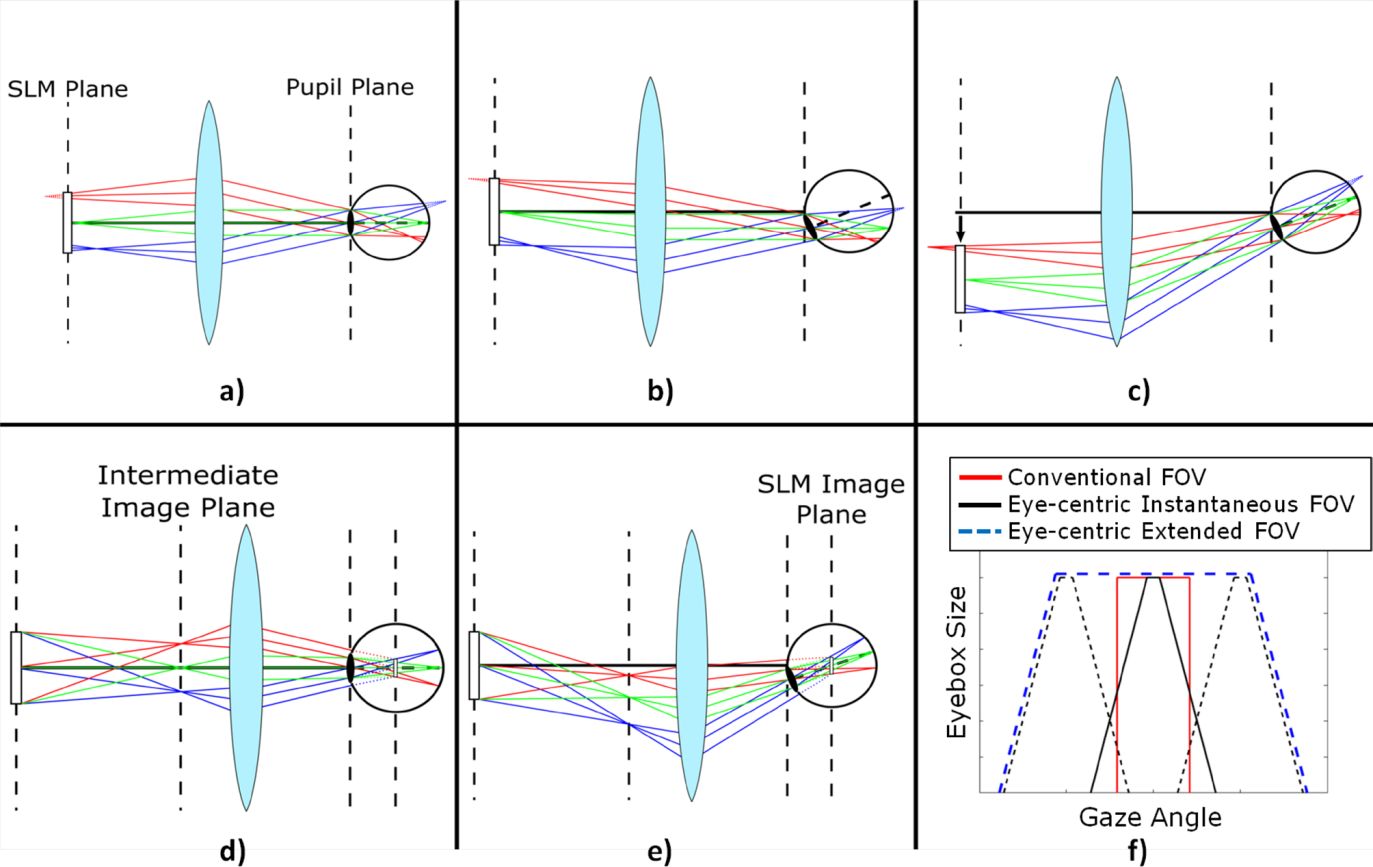
Eye-centric design concept. (**a**) Conventional display architecture where the SLM is imaged at the retina. (**b**) If the eye rotates, the SLM image shifts to the peripheral vision. (**c**) SLM movement is required to shift the image back to fovea. (**d**) In our eye-centric design the SLM is imaged at the rotation center of the eye projects a holographic image to the retina. (**e**) If the eye rotates, light from the SLM still enters through the pupil and always falls on the fovea. (**f**) Conventional displays have fixed FOV and eyebox size, therefore fixed spatial resolution across the FOV. In the eye-centric design, the eyebox size decreases towards the edges of the instantaneous FOV due to vignetting effects, which results in degrading resolution away from the foveal vision. As the instantaneous FOV follows the pupil, an extended FOV is achieved.

In conventional holographic displays, steering the FOV (i.e., keeping the image centered at the fovea as the eye rotates) is not possible by changing the CGH. As illustrated in Fig. 2a–c, eye rotation causes the displayed image to shift towards the peripheral vision. SLM or lenses need to be moved mechanically to bring the image onto the fovea. Imaging the SLM at the rotation center of the eye, which we call the eye-centric design, is at the core of our proposed architecture. Since the SLM is imaged at the rotation center of the eye, changing gaze directions are handled by changing the direction of rays via CGH as illustrated in Fig. 2d–e. The direction of the rays are altered by adding diffractive lens terms and grating terms on the CGH without any moving components. In essence, a demagnified SLM image at the center of the eye projects the holographic image onto the fovea and the eye rotations are handled by adding tilt terms to the CGH. FOV, eyebox size and spatial resolution tradeoffs for different cases are discussed in the supplementary material.

The eye-centric design has two major advantages compared to conventional holographic HWD architectures: (i) Instantaneous FOV can be digitally steered by only modifying the CGH to follow the gaze, providing a natural foveated display with no moving parts. (ii) The extended FOV is much larger than what is achievable with conventional holographic HWDs.

**Figure 3:**
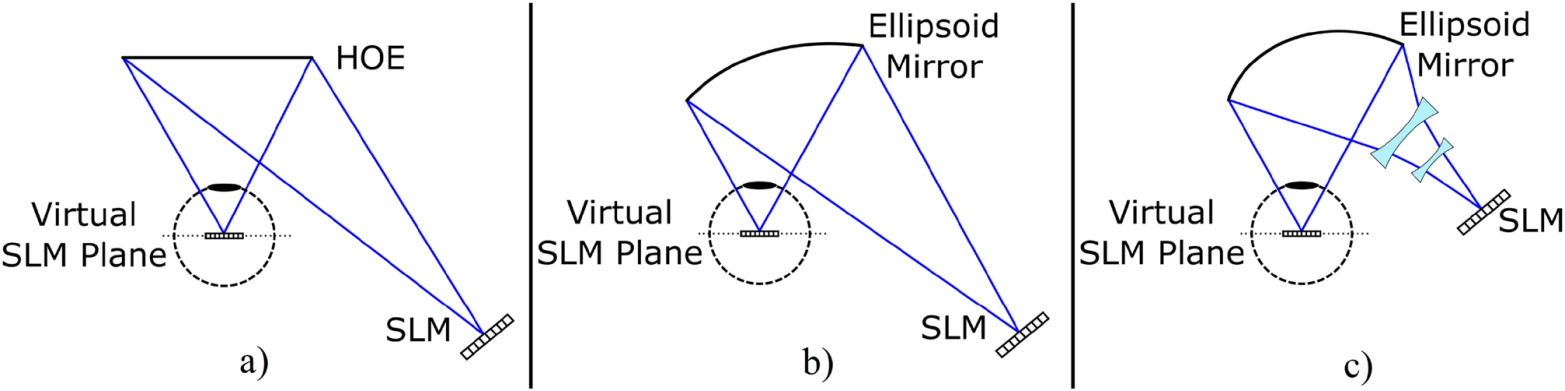
Eye-centric design implementations. SLM can be imaged at the eye-center by using a flat HOE or an ellipsoid, as illustrated in (**a**) and (**b**). Additional optics can be used to build a more compact HWD as shown (**c**).

The eye-centric design can be realized using various optical components as illustrated in Fig. 3. The key point is to image the center of the SLM to the rotation center of the eye. If the SLM image is not located at the rotation center of the eye, the pupil, the fovea, and the SLM image will not be lined up for a gaze direction other than the nominal one, which results in a reduction in the instantaneous FOV. Imaging the SLM to the center of the eye can be achieved by using a flat holographic optical element (HOE) in front of the eye that can be optimized for small form factor, but color correction remains a challenge for wide FOV. Figure 3b shows an alternative design using a section of an ellipsoid as the combiner element. An SLM that is placed at one of the foci of the ellipsoid is imaged at the second focal point. This is a simpler solution, but the magnification is not constant across the FOV. Additional imaging optics can be used to design a more compact system with balanced magnification as illustrated in Fig. 3c.

For the single ellipsoidal mirror based imaging optics case, the numerical aperture of the imaging optics is the limiting factor for the extended FOV. A larger mirror would result in a wider area where the instantaneous FOV can be steered, but it would interfere with the user’s face as the eye relief is relatively short. This would not be an issue for the temporal gaze direction, but the extremity of the change in magnification for this case results in a very small eyebox size.

Since there are no additional optics required between the eye and the ellipsoid, eye relief can be adjusted by selecting the ellipsoid parameters accordingly. If a single ellipsoid mirror with smaller eye-relief is used, the ellipse would need to be tilted more for the light from the SLM to clear the viewer’s head, and the magnification would vary more from nasal to temple direction. However, using multiple lenses, freeform lenses embedded in solid optics, and HOEs can provide good optical solutions for future implementations of the core eye-centric architecture.

## Results

**Figure 4:**
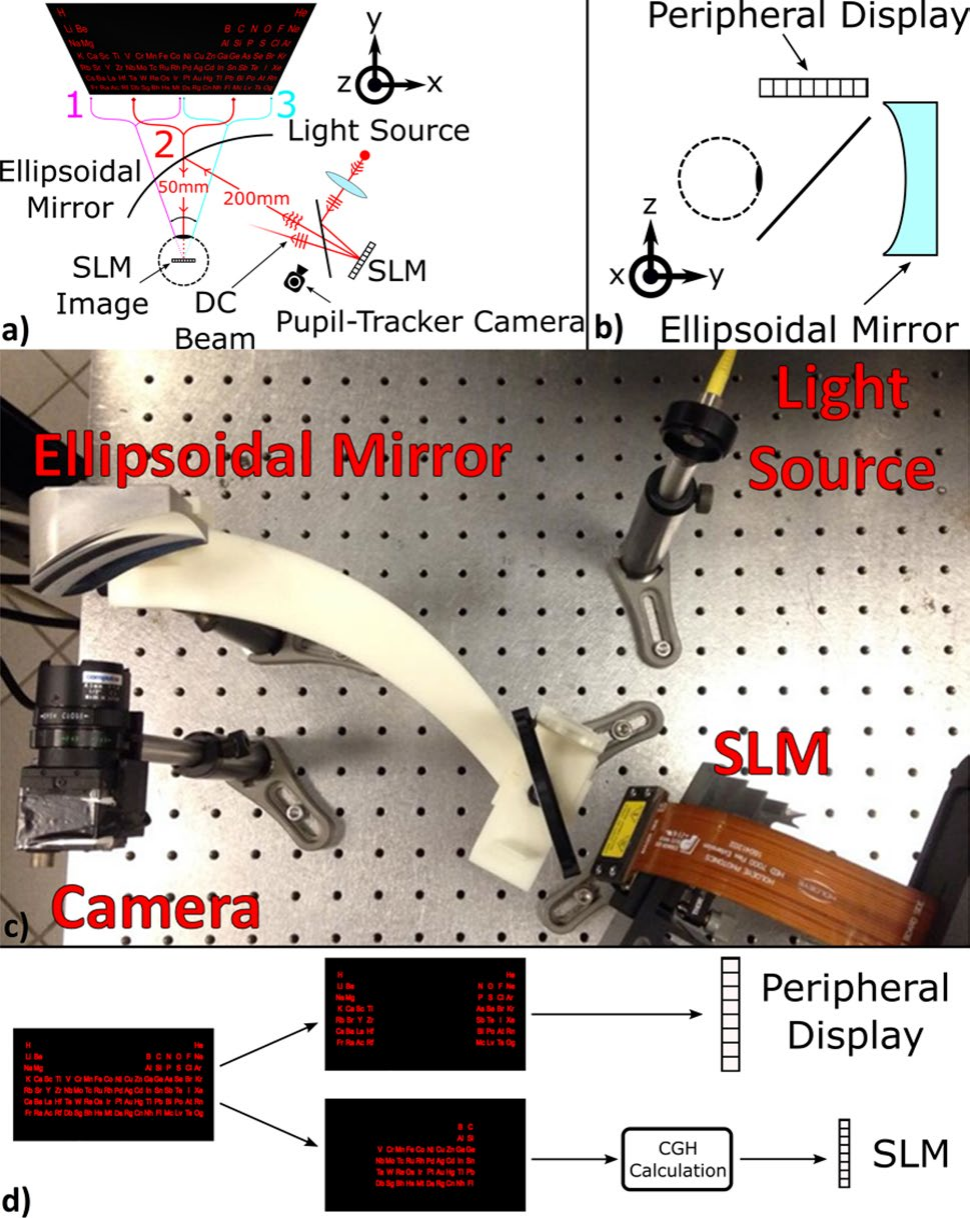
Experimental setup. (**a**) Illustration of the optical setup. (**b**) Side view illustration of the setup, where the peripheral display is shown clearly. (**c**) Experimental setup without the peripheral display and beamsplitter. (**d**) Block diagram illustrating how an input image is displayed using the SLM and the peripheral display for the nominal gaze direction.

For experimental verification of the eye-centric design, we used the imaging system in Fig. 3b. We placed the SLM at one of the focal points of the ellipsoid and the rotation center of the eye was aligned with the other focus. The ellipsoid mirror was tilted in the temple direction (towards the ear) by 17° to achieve better form factor as shown in Fig. 4.

**Figure 5:**
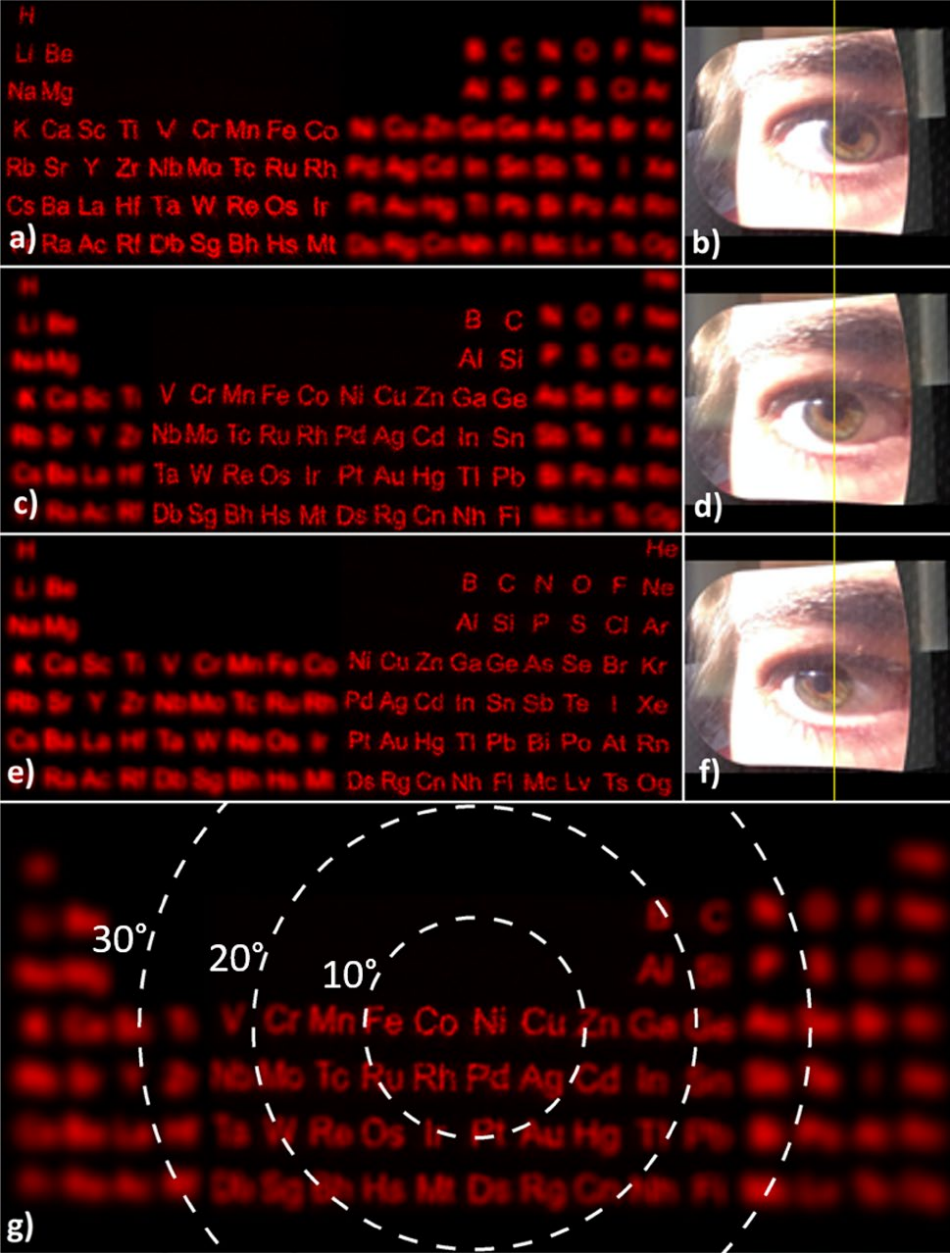
Experimental verification of foveated display. (**a,c,e**) Experimental results of the eye-centric foveated holographic display. Each row corresponds to a gaze direction shown in Fig. 4a. The periodic table is displayed by the SLM. As the eye rotates, a high-quality holographic view of different regions of the image is seen. (**b,d,f**) Corresponding pupil images captured by the camera. Note the shift of pupil center with respect to the yellow reference line due to eye rotation. (**g**) Simulation of human vision using the image in (**a**). The white rings indicate the field of view of the human eye (10°, 20° and 30° FOV). The resolution rapidly degrades away from the fovea.

Figures 4 and 5 shows the foveated display with no moving parts. A low-resolution image of the periodic table is displayed on the peripheral display at all times. The high-resolution image of the viewed section is projected onto the fovea by updating the CGH on the SLM. The gaze angle is measured by a pupil tracker camera and the holograms are updated accordingly to steer the projected image. The SLM is limited to 30 fps, therefore, dynamic hologram update with real-time pupil tracker is not possible in this system. As the eye rotates (as seen by the pupil camera images), the CGH image on the fovea remains high-resolution at all times and the peripheral display image is low-resolution and blurred, mimicking human vision. The term “high resolution image” is used to refer to the central vision image provided as a hologram by the SLM. The low resolution image is used to refer to the peripheral image on an LCD, which is placed closer than the near-point of the human eye. Our experimental results are limited to about 20 degrees FOV and show about 10cycles/degrees resolution, which is below the retinal resolution limit. The limitations are mainly due to tight alignment tolerances and imperfections in producing the elliptical mirror. The content on the LCD is masked to provide only the regions outside the FOV provided by the CGH image. We demonstrated FOV steering in the horizontal direction only, so the instantaneous FOV is 20 × 20 degrees and extended FOV is up to 40 × 20 degrees in the experiments. The range was mainly limited by tight experimental tolerances and mounting aberrations in the bench setup. Simulation results in Supplementary Fig. 2 illustrate the extended FOV up to 60 × 40 degrees.

Human vision was also simulated as seen in Fig. 5g, where only the resolution degradation of the human eye was taken into consideration. The minimal resolvable angle size was assumed to be linearly increasing with eccentricity, which is a commonly used approximation in both vision and computer graphics^24,25^. The simulation results show that the difference between the regions generated by the holographic and the peripheral displays is not completely eliminated, but it is not as severe as the difference observed in the camera images.

**Figure 6:**
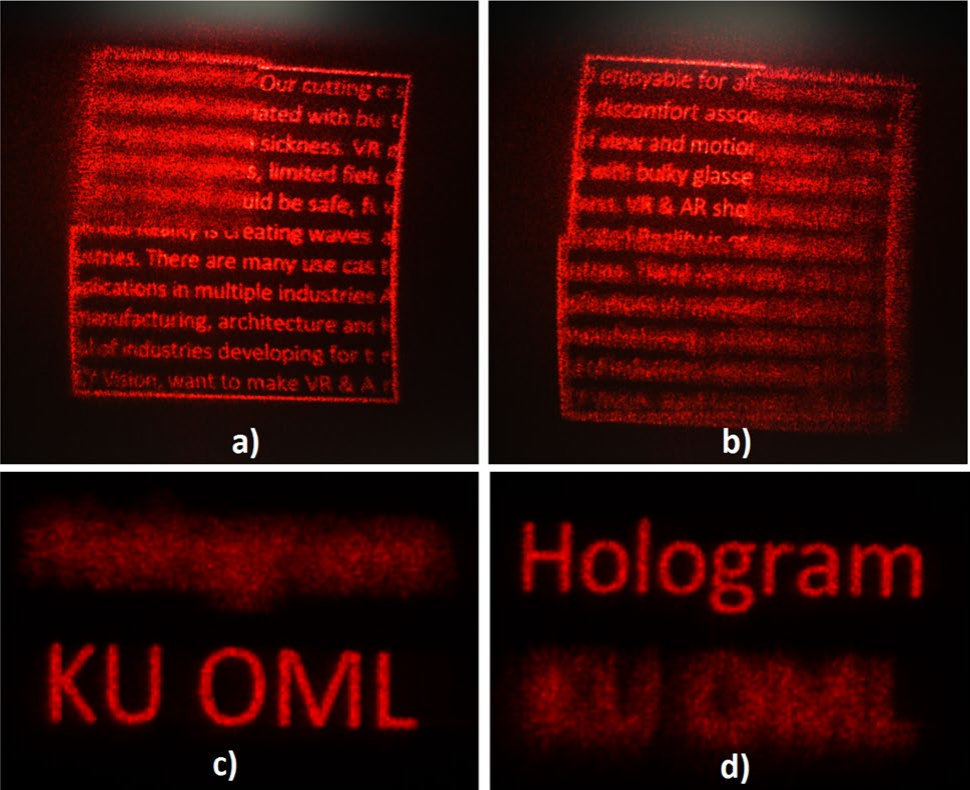
Holographic display with correct focus blur. Different sections of the text are displayed at different depths. In (**a**) and (**b**), the upper-left section of the text is displayed at 100 cm and the rest of the text is displayed at 25 cm. As the camera focus is changed, different regions appear sharp or blurred accordingly. Similarly in (**c**) and (**d**), top and bottom rows are displayed at 25 cm and 100 cm, respectively.

**Figure 7:**
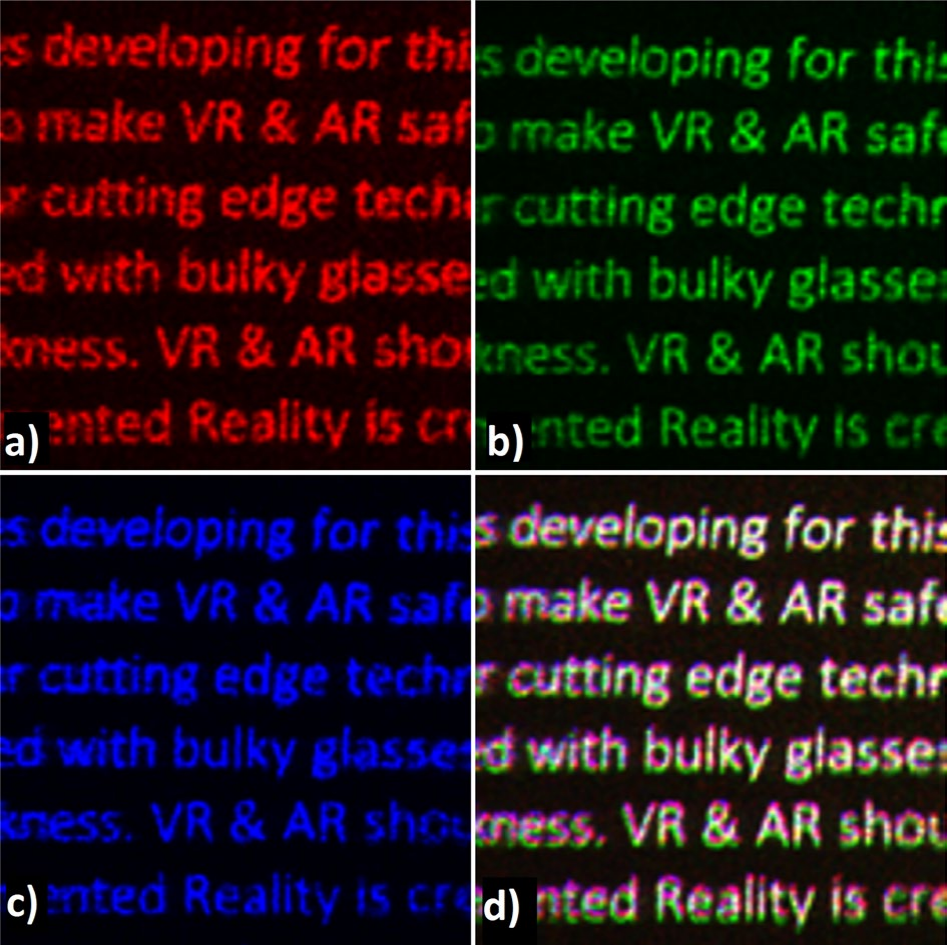
Full-color holographic demonstration. (**a–c**) show the holographic images under monochromatic illumination of wavelengths 639 nm, 515 nm, 473 nm, respectively. (**d**) Full color image with time-sequential illumination of RGB wavelengths.

Figure 6 demonstrates the capability of providing true depth information of our foveated holographic display. Objects are displayed at different depths so only the gazed depth appears sharp, while others appear blurred due to expected focus blur effect. Note that coherent blur appears sharper in camera images and smoother to the eye. We can also display color holograms by time-sequentially updating the CGH on the SLM and turning on the correct light source as seen in Fig. 7.

## Discussion

In this paper, we proposed the eye-centric approach for 3D holographic HWDs, which makes it possible to steer the instantaneous FOV within a larger extended FOV without using any moving parts or dynamic optical components. Given a reflective phase-only SLM, we have developed a design procedure for an eye-centric holographic HWD and described an algorithm to compute holograms for any optical setup as discussed in detail in the supplementary material. The experimental results verify that the proposed system is able to display 3D images in a wide, steerable FOV.

In conventional holographic HWDs, the rays from any virtual object point fill the entire eyebox. The eyebox size and resolution are constant across the FOV as shown in Fig. 2f. However, the resolution in our eye-centric architecture is not constant across the FOV. As illustrated in Fig. 2d–e, while rays from central object points fill the eyebox fully, those from the peripheral object points fill the eyebox partially, due to vignetting from the SLM. As the object points get closer to the periphery, the available eyebox size decreases gradually. This results in a degrading resolution towards the peripheral vision, mimicking the human visual system. Using this effect, it is possible to achieve an instantaneous FOV that is larger than the FOV achieved in conventional architectures as compared in Fig. 2f.

Magnification (M) is one of the key parameters of the ellipsoid for our display. Smaller magnification brings larger eyebox and FOV, but high enough magnification is needed for sufficient separation of the diffraction orders due to the pixelated structure of the SLM, which results in a small eyebox and small FOV. Therefore, an optimum point should be selected for a given SLM and desired display parameters. According to our analysis, M = 4 gives the best trade-off between eyebox size and instantaneous FOV. For detailed discussions on selecting the optimal magnification, see the supplementary material.

Experimental demonstrations were limited to an instantaneous FOV of 20 × 20 degrees. Zemax simulations show that a 28 × 28 degrees FOV is achievable with this architecture. While the FOV aspect ratio is linked to pixel aspect ratio (equal to 1 for square pixels), the resolution in each axis is linked to display aspect ratio (equal to 16:9 for our SLM). Conventional holographic display architectures can provide a static FOV of 20 × 11.25 degrees using a 4 K SLM. Therefore, our proposed architecture can effectively provide a total enhancement of 1.4 × 2.5 times improvement in horizontal and vertical axis. Furthermore, the architecture allows for steering the FOV within an extended FOV as the eye rotates. We show that 3 × 4 times improvement in horizontal and vertical extended FOV (> 10 × in extended FOV area) and a similar improvement in space-bandwidth product is achievable.

For eye-centric design to work as intended, the pupil position should be measured in real time to update the hologram on the SLM and the image on the peripheral display accordingly. We provided basic proof of concept demonstrations using off-line computed holograms and precise positioning of the camera during recording of the experimental images. Details of real-time pupil tracking^26,27^ and real time computation of CGH for generic architectures^9^ are discussed elsewhere. Fast hologram computation for eye-centric architecture will be the subject of future publications.

## Materials and methods

In our experiments, we used Holoeye GAEA as the SLM, which is a 4 K (3,840 × 2,160 pixels) LCoS phase modulator chip with 3.74 μm pixel pitch. The light source is a LASOS MCS4 series 3 wavelength fiber coupled laser with wavelengths of 473 nm, 515 nm and 639 nm for blue, green and red colors, respectively. The ellipsoid mirror in our experiments has the equation parameters a = 125 mm, b = 86.96 mm and c = 86.96 mm, as defined in the supplementary material. We cut a section of the ellipsoid that gives 60° horizontal and 40° vertical FOV. The part is fabricated using diamond turning on aluminum substrate.

In the optical setup, the RGB laser beam out of a single mode fiber is collimated by a lens and the collimated beam is reflected towards the SLM by a pellicle beamsplitter to avoid ghosts. The incident beam on the SLM is modulated by the CGH and reflected towards the ellipsoid. The ellipsoid creates the Fourier plane at the eye pupil plane and the diffraction orders are filtered-out by the pupil. The ellipsoid also images the SLM to the rotation center of the eye and a holographic image forms on the retina. A pupil tracker camera measures the pupil position and the CGH on the SLM is updated accordingly.

The 3D scene is modelled as a set of luminous points, similar to methods where 3D objects are represented by point clouds^28–30^, and the hologram corresponding to the entire scene is generated by superposing the holograms for the individual points. The hologram for a single point is computed with an algorithm based on ray-tracing, which makes use of Zernike polynomials to estimate the wavefront exiting the SLM^31–33^. As a final step, the iterative Fourier transform algorithm is employed for phase encoding^34,35^. The hologram calculation algorithm is discussed in further detail in the supplementary material.

As the fabricated ellipsoid is not transparent, a true augmented reality demonstration could not be made. In order to provide the peripheral image, we inserted a beam splitter between the eye and the ellipsoid and showed the low-resolution peripheral image on an LCD screen at 10 cm from the eye location without using any magnifier lenses in order to keep the overall complexity of the system low. The peripheral display can be located at any distance from the eye. We chose a distance of 10 cm to keep the sizes of both the peripheral display and the entire HWD bench top prototype small.

## Supplementary information

Supplementary figure 1

Supplementary figure 2

Supplementary figure 3

Supplementary figure 4

Supplementary information

## Data Availability

The data supporting the figures in this paper and other findings of this study are available from the corresponding author upon request.
