# Supplementary figures and images for: Foveated near-eye display using computational holography
Source: Sci Rep. 2020 Sep 10;10:14905. doi: 10.1038/s41598-020-71986-9 (PMC7483548; doi:10.1038/s41598-020-71986-9)

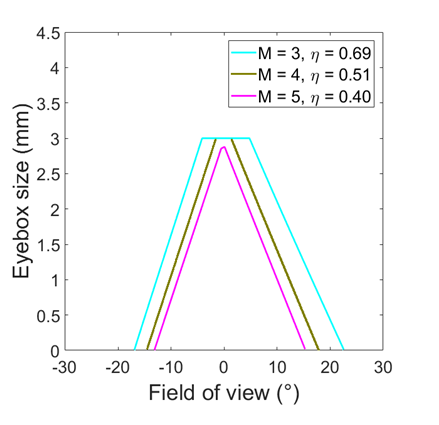

Supplement: Supplementary file 1 — Supplementary figure 1 [file 41598_2020_71986_MOESM1_ESM.tif]

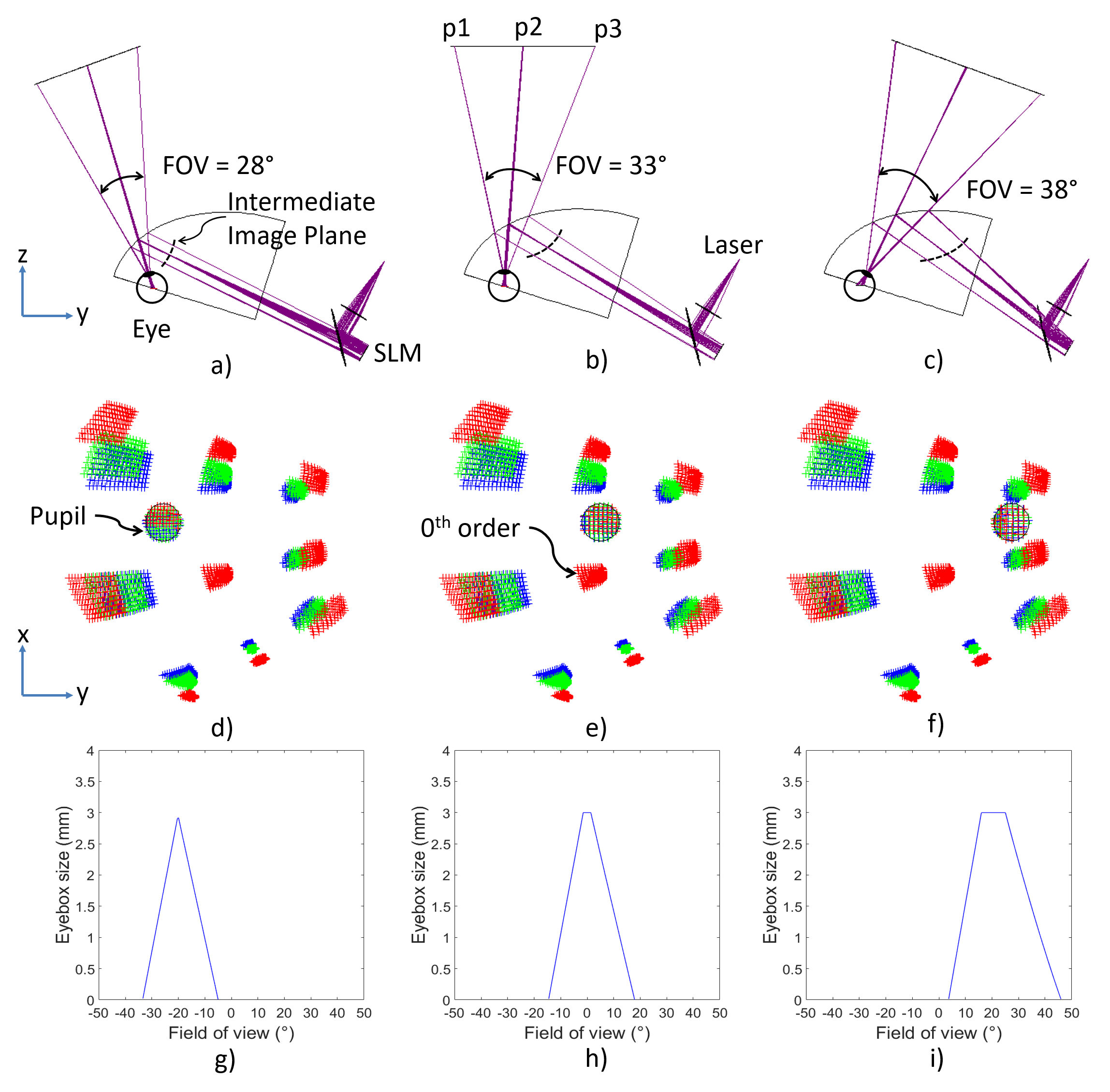

Supplement: Supplementary file 2 — Supplementary figure 2 [file 41598_2020_71986_MOESM2_ESM.tif]

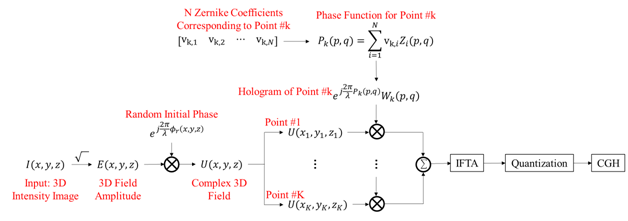

Supplement: Supplementary file 3 — Supplementary figure 3 [file 41598_2020_71986_MOESM3_ESM.tif]

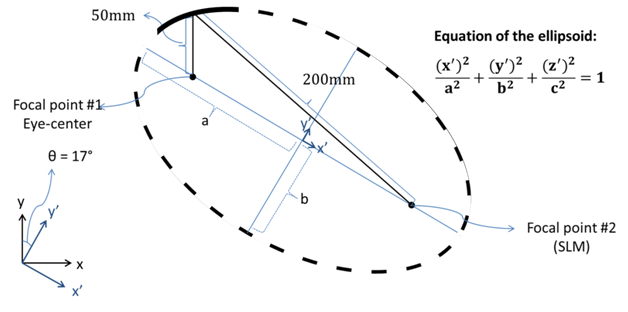

Supplement: Supplementary file 4 — Supplementary figure 4 [file 41598_2020_71986_MOESM4_ESM.tif]
