# Supplementary material for: Foveated near-eye display using computational holography
Source: Sci Rep. 2020 Sep 10;10:14905. doi: 10.1038/s41598-020-71986-9 (PMC7483548; doi:10.1038/s41598-020-71986-9)
Supplement: Supplementary file 5 — Supplementary information [file 41598_2020_71986_MOESM5_ESM.docx]

**Foveated Near-Eye Display Using Computational Holography**

**Ali Cem^1,+^, M. Kivanc Hedili^1,+^, Erdem Ulusoy^1^, Hakan Urey^1,*^**

^1^Optical Microsystems Laboratory, Koç University, Electrical and Electronics Engineering Department, Istanbul, 34450, Turkey

^+^these authors contributed equally to this work

^*^hurey@ku.edu.tr

**Undesired Beams and Spatial Filtering:**

In their current state, commercially available SLMs introduce additional challenges that must be resolved so that our proposed eye-centric holographic HWD approach can be properly realized. The pixelated structure of LCoS SLMs results in diffraction orders of both the modulated signal beam and the unmodulated illumination beam. As the pixel sizes for commercially available SLMs are typically around 4-6 microns, the angular separation between the diffraction orders is less than 10° for visible light. The unmodulated DC beam and its replicas emerging from the SLM can be filtered out at the pupil plane by introducing an angular tilt term for the signal beam and aligning it with the middle of the DC beam and its first diffraction order. Then, by designing the illumination optics so that the unmodulated beams are focused at the pupil plane, the pupil itself can filter them out along with the replicas of the signal beam. Using the paraxial approximation, it is possible to conduct a 2D analysis that will produce useful relations between the optical design parameters of the system. Doing so, the separation between the focused spots resulting from the unmodulated beams on the pupil plane, which we call the maximum eyebox size is given by

$$\begin{aligned} EB_{max}=\frac{\lambda Mr}{\Delta}, \#\left( 1 \right) \end{aligned}$$

where M is the demagnification of the imaging system for a given gaze angle, r is the distance between the center of the eye and the pupil plane, and ∆ is the pixel pitch of the SLM. In practical applications, the central part of the eyebox with size equal to that of the pupil is used for the signal beam and the area close to the edges of the eyebox is reserved for noise resulting from phase encoding, which gets filtered out by the pupil. The eyebox utilization ratio η is defined to be the ratio between the pupil size ${EB}_{p}$ and the maximum eyebox size. Under this formulation, the required demagnification for a given eyebox utilization ratio and pupil size can be found using

$$\begin{aligned} M=\frac{EB_{p}}{\eta}\frac{\Delta}{\lambda r} \#\left( 2 \right) \end{aligned}$$

Having found the demagnification of the optical system, the size of the SLM image $W_{SLM}$ at the eye center can also be calculated as

$$\begin{aligned} W_{SLM}=\frac{N\Delta}{M}, \#\left( 3 \right) \end{aligned}$$

where $N$ is the number of pixels of the SLM along the axis of interest. The size of the SLM image, the pupil size and the distance between the pupil and the eye center is sufficient to calculate the eyebox size supported by the SLM for a virtual object point at infinity. For points inside the central field-of-view $FOV_{c}$ (the field-of-view for which the eyebox size is at its peak value), the supported eyebox size $EB_{c}$, which is the maximum eyebox size, is equal to the minimum of ${EB}_{p}$ and $W_{SLM}$. The eyebox size decreases linearly for points outside $FOV_{c}$ and reaches zero for a point on the edge of the instantaneous field-of-view $FOV_{i}$, which is defined as the total field-of-view for a fixed gaze direction. The relations between the FOVs and the physical design parameters are given by the following equations:

$$\begin{aligned} FOV_{c}=\left| \frac{{EB}_{p}-W_{SLM}}{r} \right| \#\left( 4 \right) \end{aligned}$$

$$\begin{aligned} FOV_{i}=\frac{{EB}_{p}+W_{SLM}}{r} \#\left( 5 \right) \end{aligned}$$

The trapezoidal eyebox-FOV curve becomes triangular when the pupil size is equal to the SLM image size. The area under the curve, which is equal to the space-bandwidth product $SBP$ can be expressed as

$$\begin{aligned} SBP=\frac{FOV_{c}+FOV_{i}}{2}EB_{c}=N\lambda\eta\#\left( 6 \right) \end{aligned}$$

Note that for a given SLM, while the SBPs of our novel eye-centric architecture and the conventional holographic HWD architecture are the same, the eye-centric design has a larger FOV. The main difference between the two approaches in terms of their eyebox-FOV curves is that the eyebox size is constant over the FOV for the conventional approach while the eyebox size decreases gradually near the edge of the FOV for the eye-centric approach, making it possible to have a higher instantaneous FOV without changing the eyebox size for the central FOV. The eye-centric approach provides full resolution only at the fovea and the resolution degrades towards the edges of the FOV, which is consistent with the human visual system. Therefore, the eye-centric architecture results in a more efficient utilization of the available space-bandwidth product.

Limiting the analysis to the horizontal FOV, designing for a full HD SLM with a pixel pitch of 4.5 microns and assuming a pupil size of 3 mm with an eyebox utilization ratio of 0.8, it is apparent that the conventional approach cannot have an instantaneous FOV of more than 15°. On the contrary, it is possible to reach an instantaneous FOV of 25° using the eye-centric approach.. Selecting the demagnification high enough so that the unmodulated beams and the diffraction orders are separated at the pupil plane makes the SLM image so small that the supported eyebox size is 2.8 mm even at the center of the FOV. Furthermore, the central FOV is only slightly larger than 2°, which is significantly smaller than the FOV for human central vision. In order to overcome these problems while preserving the eye-centric property of the system, an SLM with smaller pixel pitch and larger pixel count is required. Using a 4K SLM with a pixel pitch of 3.74 microns with a demagnification of 2.6 resolves all of the previously discussed issues: the central FOV exceeds 10° and the corresponding eyebox size reaches 3 mm. In addition, the overall instantaneous FOV is higher than 30°.

Smaller magnification brings larger central and instantaneous FOV, but high enough magnification is needed for sufficient separation of the diffraction orders, which is required to obtain a large clear eyebox. As the magnification is decreased, the space-bandwidth utilization ratio gets closer to 1 as the EB_max_ gets closer to the pupil size, which is undesired. Therefore, an optimal point should be selected for a given SLM and desired display parameters. As seen in Supplementary Fig. 1, M = 4 gives the best trade-off. The central eyebox size is not lower than the pupil size as in the case of M = 5 and $\eta$ is not very close to 1, which is the case for M = 3.


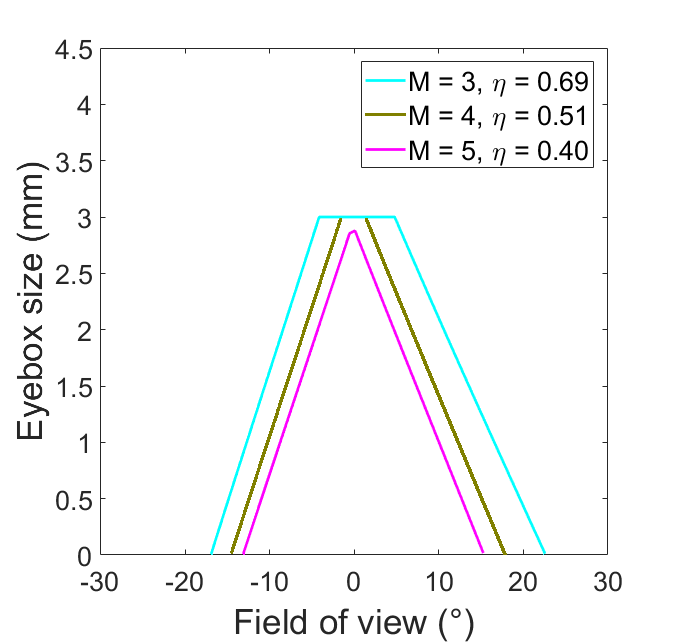


Supplementary Figure 1 Ellipsoids with different nominal magnifications were analyzed. We chose M = 4 as it provides a good trade-off between the eyebox size and the eyebox utilization ratio η. As each point on the ellipsoid has a different focal length, the magnification across the FOV is non-uniform. This results in an asymmetrical trapezoid.

We designed and simulated the ellipsoid with a nominal magnification M = 4 using Zemax and calculated the corresponding FOV-Eyebox size curves in MATLAB. As seen in Supplementary Fig. 2a-c, the instantaneous FOV varies for different gaze angles due to non-uniform magnification across the ellipsoid. Moreover, this non-uniformity introduces a significant distortion to the wavefront as seen in the spot diagram at the pupil plane in Supplementary Fig. 2d-f. Spot diagrams show the diffraction orders of the illumination beam for RGB colors. The clear region between the orders is the eyebox of our display. The eyeball diameter is assumed to be 20mm in the simulations. A pupil with 3 mm diameter filters out the diffraction orders so that the image can be seen clearly. Simulation starts from a point light source and illustrates that +/- 20deg eye-rotation in the horizontal axis is easily achievable by simply adding a linear phase term on the computed phase hologram pattern to move it across the pupil plane. Due to varying magnification across the eyebox, diffraction orders produce aberrated spots across the pupil plane. Supplementary Fig. 2g-i show the corresponding eyebox-FOV curves for each gaze angle. Clear eyebox size decreases, hence the instantaneous FOV increases from nasal to temporal gaze angles.

Since the effective focal length is not constant on the ellipsoid, the magnification is non-uniform across the combiner surface. We defined the nominal magnification as the ratio of the distance from eye-center to ellipsoid and the distance from the ellipsoid to the center of the SLM for 0° gaze angle. This non-uniform magnification brings asymmetry to the trapezoid in eyebox size-FOV curve (Supplementary Fig. 1).


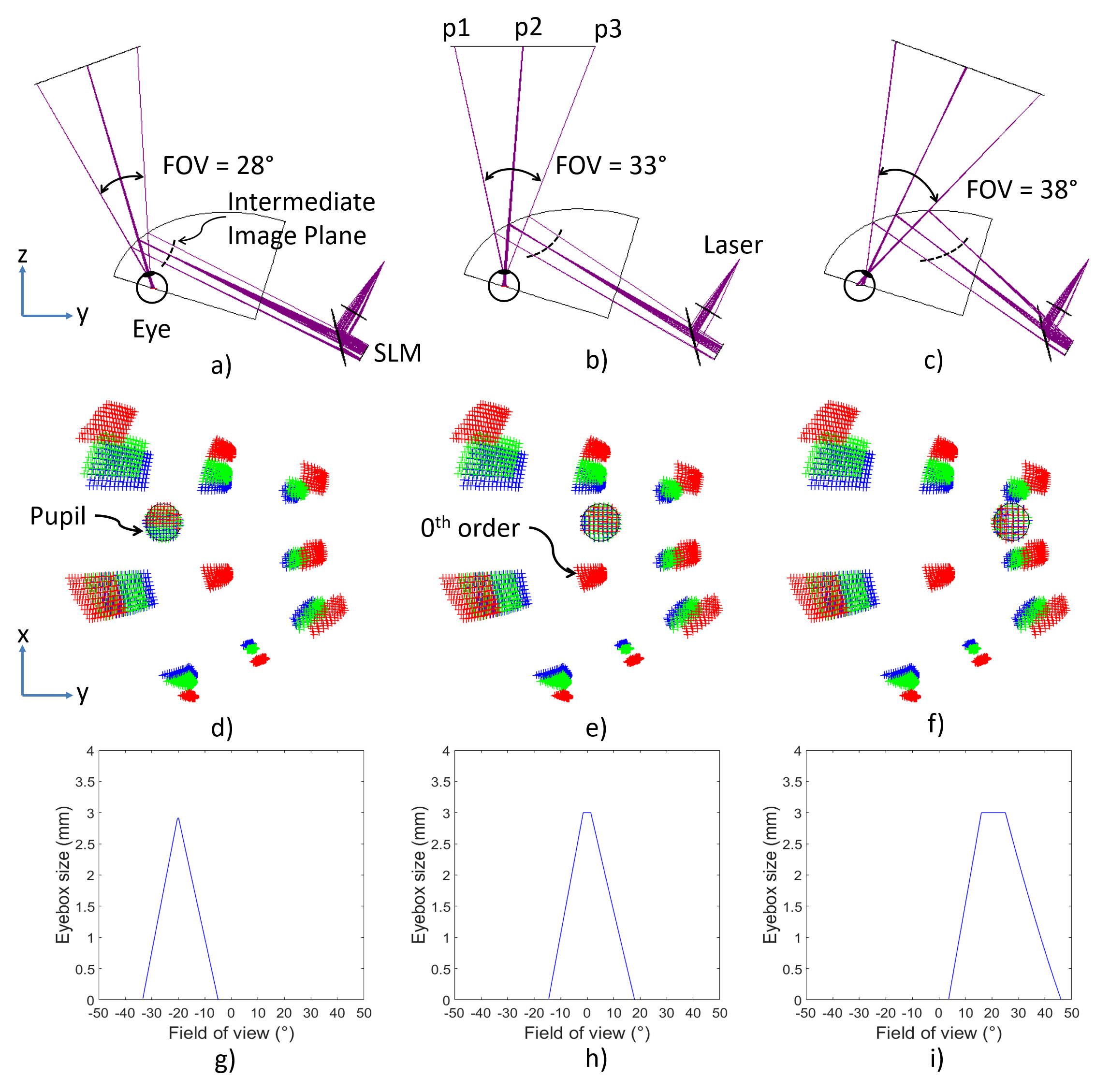


Supplementary Figure 2 Zemax simulation of the eye-centric system using an ellipsoid. Each column shows different gaze directions (-20°, 0° and 20°): The instantaneous FOV at the top, the eyebox profile in the middle and the corresponding eyebox-FOV curve at the bottom.

In our calculations the instantaneous FOV boundary is defined as the point where only the edge of the virtual object beam barely enters the eye pupil, as seen in points p1 and p3 in Figure 2. It is important to note that the instantaneous FOV also depends on the size of the pupil, which may vary due to changes in ambient brightness. More specifically, if the pupil size is smaller than expected, the instantaneous FOV will be decreased. In order to minimize this effect, we assumed a small pupil size of 3 mm during optical design and hologram computation. In contrast, if the pupil size is larger than the clear eyebox size, undesired beams may enter through the pupil degrading the image quality and contrast. One advantageous feedback mechanism is that as the bright unmodulated beams enter through the pupil, the pupil gets smaller. Furthermore, in a future implementation, it is possible to filter out the unmodulated beams at an intermediate image plane. This way, the risk of high intensity diffraction spots falling inside the pupil as the eye rotated would be eliminated as well.

**Hologram Calculation for Eye-Centric Design**

Conventional CGH computation algorithms typically use the angular spectrum method to numerically propagate the optical field from a 3D scene to the hologram plane where the SLM is located^1^. Optical components used in conventional holographic HWDs such as beamsplitters or lenses are approximated using paraxial models with high accuracy, which makes it possible to use an in-line model for the entire setup. In our experimental setup using the eye-centric architecture, an off-axis ellipsoidal mirror is required to image the SLM to the eye center, which cannot be modelled as a paraxial lens due to its varying magnification across the FOV. Therefore, we have developed an algorithm based on ray tracing to calculate CGHs, where the 3D scene is considered to be a set of luminous points emitting spherical waves, similar to how the 3D scene is decomposed in the wave-propagation based methods where objects are represented by point clouds ^2–4^.

In order to compute the hologram for a point object, rays must be traced using ray tracing software back from the virtual image point to the SLM, where a 2D phase function approximating the wavefront can be fit. A 66 by 66 uniform grid of rays were emitted from the object, filling the pupil entirely. By placing a retroreflector in place of the pupil, the rays were directed back to the reflective ellipsoidal surface, which reflects them to the SLM. The points where the rays hit the SLM are denoted by $(p_{i},q_{i})$, where $p$ and $q$ correspond to the coordinates on the horizontal and vertical axes passing through the center of the SLM, respectively. The area on the SLM covered by the rays is given by $w(p,q)$, which is equal to 1 where the rays can reach the SLM and 0 otherwise. The angle between the ray with index $i$ and the p-axis is defined to be $\alpha_{i}$ and the angle between the same ray and the q-axis is defined to be $\beta_{i}$. The phase function $\phi\left( p,q \right)$ must satisfy the following conditions so that it accurately describes the wavefront on the SLM required for the reconstruction of the point object:

$$\begin{aligned} \frac{\partial}{\partial p}\phi\left( p_{i},q_{i} \right)=\sin\left( \alpha_{i} \right) \#\left( 7 \right) \end{aligned}$$

$$\begin{aligned} \frac{\partial}{\partial q}\phi\left( p_{i},q_{i} \right)=\sin\left( \beta_{i} \right) \#\left( 8 \right) \end{aligned}$$

Many different methods for finding such phase functions have been developed, especially in the field of optical metrology^5–7^. One possible way of finding such a phase function is to assume that it is in the following form:

$$\begin{aligned} \phi\left( p,q \right)\approx\phi_{z}\left( p,q \right)=\sum_{i=1}^{N} a_{i}Z_{i}\left( p,q \right), \#\left( 9 \right) \end{aligned}$$

where $Z_{i}$ is the Zernike polynomial with Noll index $i$ and $N$ is the number of Zernike polynomials used to approximate the phase function. Under this formulation, the problem of fitting a phase function is equivalent to finding $N$ Zernike coefficients that best satisfy the phase constraints. The Zernike coefficients were computed by finding the minimum norm least squares solution to the overdetermined system. Simulations have shown that choosing $N=10$ results in reconstruction errors less than that of a diffraction-limited optical system.

Having computed the phase function $\phi(p,q)$, the full-complex hologram for a point object is given by $H\left( p,q \right)=\sqrt{I}e^{j\frac{2\pi}{\lambda}\phi(p,q)}w(p,q)$, where $I$ denotes the optical intensity of the object. In order to compute the full-complex hologram of the entire 3D content to be displayed, one can simply treat it as a collection of points located at the centers of the voxels making up the 3D content. The full-complex hologram is then expressed as the summation of all individual point object holograms.

As a final step, the full-complex hologram must be encoded to a phase pattern so that it can be displayed on a phase-only SLM. The complex function obtained by superposing the holograms for all virtual object points is approximated using a phase-only hologram computed using the Iterative Fourier Transform Algorithm (IFTA) with 10 iterations, which is a well-known method for encoding complex CGHs into phase or amplitude CGHs^8,9^.

The proposed algorithm for CGH computation includes ray tracing and surface fitting for every point on the 3D content, which results in very high computational cost. In practice, we approximated the holograms for some object points using the holograms of nearby object points to accelerate hologram calculation.


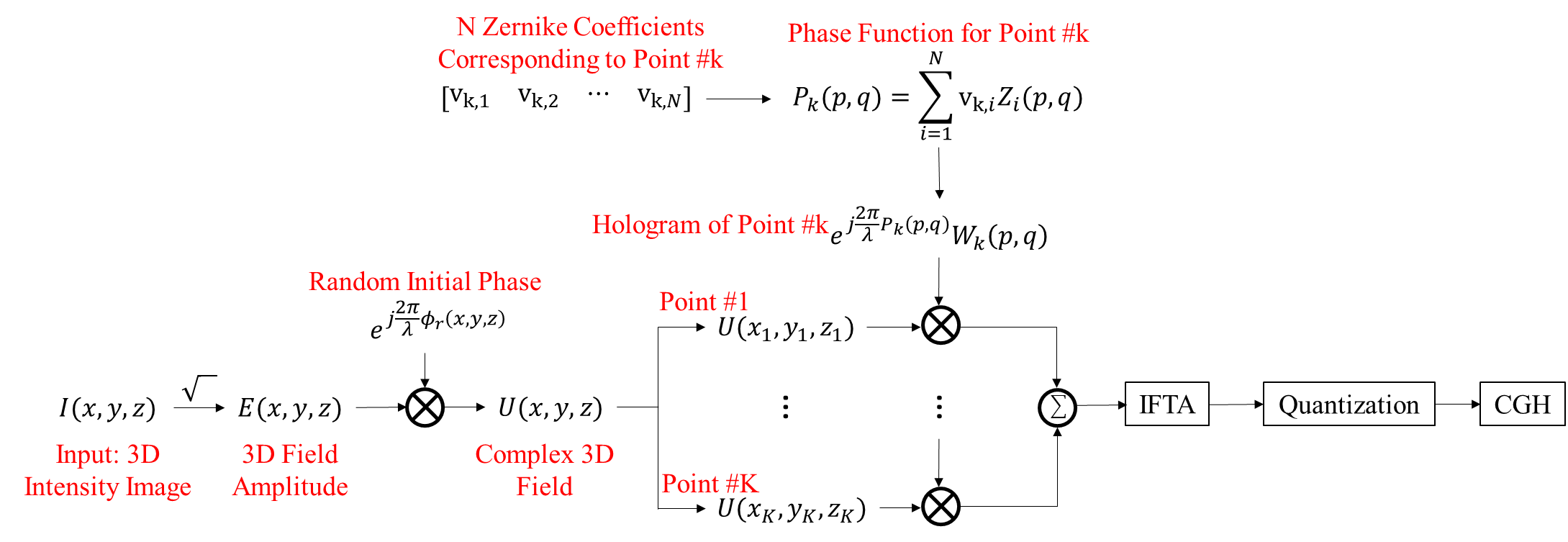


Supplementary Figure 3 Block diagram summarizing the hologram computation algorithm. Note that $\boldsymbol{p}$ and $\boldsymbol{q}$ correspond to the horizontal and vertical coordinates on the SLM and IFTA is the Iterative Fourier Transform Algorithm used for phase encoding^8,9^

**Ellipsoid Parameters:**


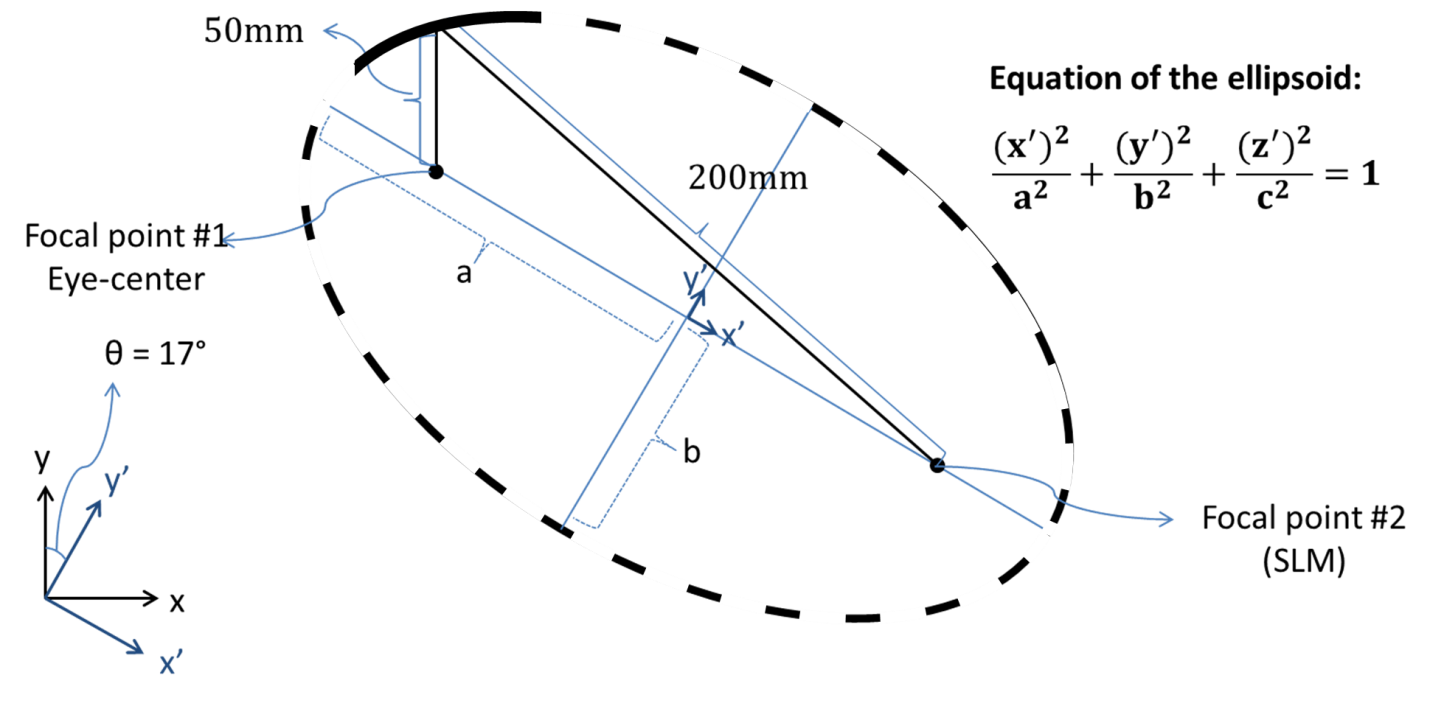


Supplementary Figure 4 Geometrical parameters for the exact shape and orientation of the ellipsoid. The ellipsoid parameters are: a = 125mm, b = 86.96mm, c = 86.96mm.

**References**

1. Park, J. H. Recent progress in computer-generated holography for three-dimensional scenes. J. Inf. Disp. (2017). doi:10.1080/15980316.2016.1255672

2. Lucente, M. E. Interactive computation of holograms using a look-up table. J. Electron. Imaging (1993). doi:10.1117/12.133376

3. Shimobaba, T., Nakayama, H., Masuda, N. & Ito, T. Rapid calculation algorithm of Fresnel computer-generated-hologram using look-up table and wavefront-recording plane methods for three-dimensional display. Opt. Express (2010). doi:10.1364/oe.18.019504

4. Kim, S.-C., Dong, X.-B., Kwon, M.-W. & Kim, E.-S. Fast generation of video holograms of three-dimensional moving objects using a motion compensation-based novel look-up table. Opt. Express (2013). doi:10.1364/oe.21.011568

5. Shi, Y., Cao, H., Gu, G. & Zhang, J. Technique of applying computer-generated holograms to airborne head-up displays. in Holographic Displays and Optical Elements II 3559, 108–112 (2003).

6. Cao, H., Sun, J. & Chen, G. Bicubic uniform B-spline wavefront fitting technology applied in computer-generated holograms. in 2nd International Symposium on Advanced Optical Manufacturing and Testing Technologies: Advanced Optical Manufacturing Technologies (2006). doi:10.1117/12.674234

7. Li, S. et al. A practical method for determining the accuracy of computer-generated holograms for off-axis aspheric surfaces. Opt. Lasers Eng. (2016). doi:10.1016/j.optlaseng.2015.08.009

8. Fienup, J. R. Iterative Method Applied To Image Reconstruction And To Computer-Generated Holograms. Opt. Eng. (1980). doi:10.1117/12.7972513

9. Wyrowski, F. & Bryngdahl, O. Iterative Fourier-transform algorithm applied to computer holography. J. Opt. Soc. Am. A (1988). doi:10.1364/josaa.5.001058
